# A method for named entity normalization in biomedical articles: application to diseases and plants

**DOI:** 10.1186/s12859-017-1857-8

**Published:** 2017-10-13

**Authors:** Hyejin Cho, Wonjun Choi, Hyunju Lee

**Affiliations:** School of Electrical Engineering and Computer Science, Gwangju Institute of Science and Technology, 123 Chemdangwagi-ro, Buk-gu, Gwangju, Republic of Korea

**Keywords:** Text mining, Named entity recognition, Entity name normalization, Disease names, Plant names, Neural networks

## Abstract

**Background:**

In biomedical articles, a named entity recognition (NER) technique that identifies entity names from texts is an important element for extracting biological knowledge from articles. After NER is applied to articles, the next step is to normalize the identified names into standard concepts (i.e., disease names are mapped to the National Library of Medicine’s Medical Subject Headings disease terms). In biomedical articles, many entity normalization methods rely on domain-specific dictionaries for resolving synonyms and abbreviations. However, the dictionaries are not comprehensive except for some entities such as genes. In recent years, biomedical articles have accumulated rapidly, and neural network-based algorithms that incorporate a large amount of unlabeled data have shown considerable success in several natural language processing problems.

**Results:**

In this study, we propose an approach for normalizing biological entities, such as disease names and plant names, by using word embeddings to represent semantic spaces. For diseases, training data from the National Center for Biotechnology Information (NCBI) disease corpus and unlabeled data from PubMed abstracts were used to construct word representations. For plants, a training corpus that we manually constructed and unlabeled PubMed abstracts were used to represent word vectors. We showed that the proposed approach performed better than the use of only the training corpus or only the unlabeled data and showed that the normalization accuracy was improved by using our model even when the dictionaries were not comprehensive. We obtained F-scores of 0.808 and 0.690 for normalizing the NCBI disease corpus and manually constructed plant corpus, respectively. We further evaluated our approach using a data set in the disease normalization task of the BioCreative V challenge. When only the disease corpus was used as a dictionary, our approach significantly outperformed the best system of the task.

**Conclusions:**

The proposed approach shows robust performance for normalizing biological entities. The manually constructed plant corpus and the proposed model are available at http://gcancer.org/plant and http://gcancer.org/normalization, respectively.

**Electronic supplementary material:**

The online version of this article (doi:10.1186/s12859-017-1857-8) contains supplementary material, which is available to authorized users.

## Background

With the rapid accumulation of biomedical articles, developing accurate and efficient text-mining techniques for extracting knowledge from articles has become important. In the text-mining, named entity recognition (NER) is an important element. Named entities are meaningful real-world objects in predefined specific domains, and they are presented as single words or multi-word phrases in texts. NER involves identifying both predefined entities as well as the domain of the entities or the entity types from informal texts [1]. After single words or multi-word phrases in texts have been recognized, the next step is named entity normalization by assigning suitable identifiers to recognized entities. For general entities, several natural language processing (NLP) studies, such as assigning entities to relevant Wikipedia abstracts or corresponding nodes in knowledge base, have been performed [2–4].

In biomedical articles, named entity normalization is challenging because many biological terms have multiple synonyms and term variations, and they are often referred to using abbreviations [5]. To resolve these ambiguities, several NER and normalization studies have been conducted for several entity types such as biological entities (genes, proteins, diseases, and disorders) and chemical entities (drugs and compounds) [6–9]. The Critical Assessment of Information Extraction in Biology (BioCreative) organized biomedical NLP challenges. One of the subtasks in BioCreative V was NER and normalization for disease names [10].

Although machine-learning (ML) approaches have been used for normalization, most normalization tools rely on the accuracy of domain-specific dictionaries or rules. This is because biological entities (1) have many synonyms; (2) are often referred to using abbreviations; (3) are described by phrases; and (4) are mixtures of alphabets, figures, and punctuation marks. The ProMiner [11] system follows a dictionary-based approach based on an approximate string-matching method; it was designed to detect and normalize gene and protein names. This system uses preprocessed dictionaries that include biological entities with known synonyms. MetaMap [12] was developed to improve the retrieval of relevant MEDLINE citations. This program maps biological entities to concept identifiers in the Unified Medical Language System (UMLS) Metathesaurus. GenNorm [7] and GNAT [8], which are used for gene name normalization, and ChemSpot [9], which is used for chemical name normalization, also normalize entities that were extracted by their own dictionary components. Gimli [13] is an NER tool designed to recognize the names of various biomedical entities. Because Gimli only performs NER, its functionalities are integrated into Neji [14] for providing general normalization based on prioritized dictionaries. Lee et al. [15] achieved a highest F-score of 86.46% for disease NER and normalization among 16 teams in BioCreative V. They used a dictionary-lookup approach based on the priority of dictionaries they assigned. Moara [6] recognized gene and protein mentions using a hybrid methodology for normalization; the normalization task consists of flexible matching and ML-based matching strategies. Flexible matching is accomplished by exact matching from dictionaries; ML-based matching follows a feature-based approach such as prefix/suffix, bigram/trigram similarity, and string/shape similarity. tmChem [16] applied a rule-based approach for concept normalization that converts identified mentions from articles to lexical variations such as lowercasing and removing whitespace and punctuations, and then maps them to specific database identifiers.

Unlike previous studies, DNorm [17] uses pairwise learning to normalize disease names; it assigns mentions in the text to proper concept names in a controlled vocabulary, where a mention and a concept name are represented as a vector. DNorm outperformed MetaMap and Lucene when it was trained and tested using the National Center for Biotechnology Information (NCBI) disease corpus [18]. However, because the vector consists of tokens appearing in mentions or concept names, tokens not appearing in a labeled data set might not be normalized properly. Thus, the importance of the labeled data set and predefined dictionaries, including synonym and abbreviation dictionaries, is emphasized, and it requires domain-specific dictionaries for normalization.

To some extent, the reliance on dictionaries can be reduced by understanding words at the semantic level. Word semantics are better understood within the context of these words, which are represented by the surrounding words to the left or right. For example, sentences similar to “The standard systemic treatment for prostate cancer (PCa) is androgen ablation, which causes tumor regression by inhibiting activity of the androgen receptor (AR). (PubMed ID: 18593950)” and “AR remains important in the development and progression of prostate cancer. (PubMed ID: 15082523)” are frequently repeated in biomedical texts. This allows us to infer that “prostate cancer”, “androgen receptor”, and “AR” are related words in their semantics.

Rumelhart et al. [19] represented words in a vector space, where similar words are located close together. Recently, neural-network-based approaches have been developed for word representations; these methods are useful for identifying word similarities [17]. These methods have become popular because word representation can be learned from a large amount of unlabeled data. Deep learning approaches using a large amount of unstructured data have attracted much attention [20], and they have been applied to many NLP problems with considerable success. Lample et al. [21] utilized a long short-term memory (LSTM) architecture and character-based word representations for the NER task. Ma et al. [22] proposed a neural network architecture that combines bidirectional LSTM, convolutional neural networks, and conditional random fields for the sequence labeling tasks, including part-of-speech tagging and NER. To evaluate the proposed NER system, they used the English data set from the CoNLL 2003 shared task [1]. However, these studies were not extended to the normalization task.

In this study, we propose a method for normalizing biological entities, for example, disease names and plant names, by representing words in continuous vector spaces using neural networks. We combine a dictionary-based approach and word representations using a training corpus and unlabeled PubMed abstracts to incorporate the contexts of words. We compared our new method to DNorm to normalize disease names with and without an abbreviation dictionary. We also applied our approach for normalizing plant mentions, which does not have an abbreviation dictionary. Without an abbreviation dictionary, this approach showed good performance for normalizing biological entities.

## Methods

### Data resources

#### Entity dictionary

##### Disease name dictionary

For the disease name dictionary, we used MErged DIsease vocabulary (MEDIC) [23] that combines the Diseases branch of the National Library of Medicine’s Medical Subject Headings (MeSH) and the Online Mendelian Inheritance in Man (OMIM). MeSH is a controlled vocabulary that includes synonyms in a hierarchical tree structure ranging from 16 general categories (e.g., Neoplasms) to more specific ones (e.g., Retinoblastoma) across 13 hierarchical levels. This hierarchy provides a way to navigate from higher to specific levels so that the relationships between diseases can be found. To merge the disease names in the two dictionaries, the terms under the Diseases branch was used. OMIM is a well-known resource for human genetic diseases. OMIM, unlike MeSH, is a flat list of different concepts such as phenotypes and genes, and it does not provide connections between similar diseases. MEDIC is a disease dictionary that combines the strengths of MeSH and OMIM, and it provides disease information, including disease names, concept identifiers (IDs), definitions of the diseases, information about parent nodes, and synonyms. MEDIC contains around 9 700 disease names and 67,000 synonyms.

##### Plant name dictionary

In this study, the term “plants” refers to a wide range of organisms, including trees, shrubs, and primitive plants, such as fungi, mosses, algae, and lichens. For thousands of years, plants have been valued for their medicinal and healthful qualities. Various scientific and common names are used for plants, because plant names have been derived from several civilizations (e.g., Greek and Chinese), and plants have evolved into various structures. Compared to other biological entities such as genes or proteins, for which several normalization studies have been performed, few studies on plant name normalization have been performed. To normalize plant names, we need a well-organized dictionary of plant identifiers. We extracted a viridiplantae ontology for plants from the NCBI Taxonomy database [24] that consists of NCBI taxonomy IDs, scientific names, synonyms, and hierarchical taxonomic information. The NCBI taxonomy database indexes over 150,000 viridiplantae that are constructed from whole, partial, or phonetically spelled organism names, and it provides information about organisms that are commonly used in biological research [25].

#### Corpus

Disease and plant corpora were used for training and testing normalization models. Table 1 shows the size of the corpora used in this study.

**Table 1:** NCBI disease corpus and our plant corpus

Data set	Abstracts	Total	Unique	Unique
		disease	disease	concept
		mentions	mentions	IDs
Disease training set	592	5145	1170	670
Disease development set	100	787	368	176
Disease test set	100	960	427	203
Total	792	6892	2136	790
Plant training set	128	2647	1543	1143
Plant development set	40	709	400	329
Plant test set	40	629	427	298
Total	208	3985	2370	1770

##### Disease corpus

For diseases, the NCBI disease corpus [18] was used in the present study. This corpus consists of 793 PubMed abstracts, 6 892 disease mentions, and 790 unique disease concepts using disease terms in MEDIC [23]. Preannotation was performed using PubTator [26]. After this step, the abstracts were manually annotated by 14 annotators. Finally, the annotated abstracts were curated by biomedical experts. The annotated abstracts consist of a training set, a development set, and a test set; these were respectively used to construct the models, set the hyperparameters in normalization models, and evaluate the models.

##### Plant corpus

For plants, we manually constructed training, development, and test sets because no appropriate corpus specific for plants is available. From 208 abstracts with 19 mentions per abstract, a total of 3 985 mentions were extracted and then mapped into concepts in the NCBI taxonomy database. Two annotators participated in constructing the corpus; their inter-annotator agreement (IAA) scores were 0.985 and 0.889 for plant name recognition and normalization, respectively, suggesting a high level of agreement. Details about the annotations, including the curator guidelines and IAA, are provided in the Additional file 1.

#### Abbreviation dictionary

In biomedical articles, long disease names occur many times, and they are often referred to using acronyms and other shorthand. However, a general rule for using acronyms does not exist, different abbreviations are often used for the same names, and some authors even create new acronyms. Therefore, two different words written in the same paragraph may indicate the same entities, or two different diseases may be written using the same word. For example, “Angelman Syndrome” and “Ankylosing Spondylitis” are both abbreviated as “AS”. Therefore, resolving abbreviations is an important issue in NER research. DNorm [17] used their own abbreviation dictionary to solve the problem of acronym normalization.

**Fig. 1:**
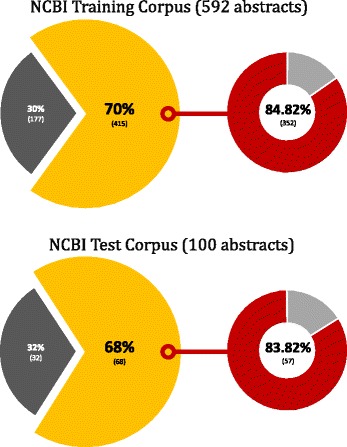
Comparison between the NCBI disease corpus and the abbreviation dictionary. The upper and the lower pie charts represent the NCBI training corpus and the NCBI test corpus, respectively. The dark gray parts represent abstracts in which disease names are not abbreviated, and yellow parts represent abstracts that contain at least one disease name abbreviation. Among the abstracts in yellow, the red parts represent abstracts with disease abbreviation information in the abbreviation dictionary, and the gray parts represent abstracts that contain at least one disease name abbreviation that is not included in the abbreviation dictionary

For disease names, we used the abbreviation dictionary provided by DNorm. It consists of PubMed IDs, disease acronyms, and original long words. However, this dictionary is optimized for the NCBI disease corpus. As shown in Fig. 1, out of the 592 abstracts in the training corpus, 415 had abbreviations for disease names, 84% of which are in the dictionary. Similarly, out of the 100 abstracts in the test corpus, 68 had abbreviations for disease names, 83% of which are in the dictionary. In addition, although a well-constructed dictionary of disease abbreviation exists, dictionaries of other biological entities such as plant abbreviation names do not exist. Thus, when we compared our approach to DNorm, we measured performances with and without this abbreviation dictionary. For plant names, we did not use an abbreviation dictionary because no dictionary is available.

### Training a normalization model

**Fig. 2:**
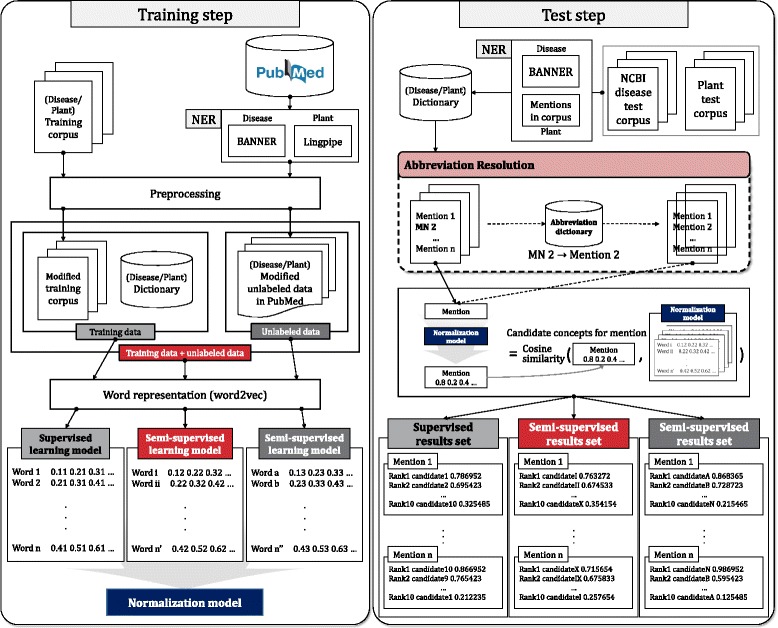
A schematic of the proposed approach

Figure 2 shows an overview of the training and test steps in our approach. In the training step, abstracts in the NCBI disease corpus and plant corpus and unlabeled data are used to construct the normalization model. In this study, the unlabeled data include a set of abstracts (or sentences) from which disease and plant names were extracted using NER tools. Note that they are considered unlabeled data because the disease and plant names were not normalized. The disease and plant names in the unlabeled PubMed abstracts were extracted using BANNER [27] and LingPipe [28], respectively. Then, we modified the training corpus and the unlabeled data from PubMed using synonyms and concepts of biological entities in the dictionaries. Finally, we represented all words in the modified training data sets and unlabeled data from PubMed in the vector space using Word2Vec [29]. The details are described in the following subsections.

#### Incorporating information in training data sets

We describe how information in the entity dictionaries and the training corpus are incorporated before we construct word vectors for all tokens in the training corpus, unlabeled data, and entity dictionaries. Throughout this paper, the names for biological entities in the sentences are called mentions.

We replaced mentions in the sentences from the training corpus and unlabeled data with synonyms in the dictionary and concepts in the training corpus. For example, if “cancer” was mentioned in a sentence, new sentences were created in which “cancer” was replaced by its synonyms such as “neoplasms”, “tumor”, “tumors”, “tumour”, or “tumours”. We also added stemming variations of disease names. The lexical variations were obtained with a stemming analyzer in Apache Lucene, which implements the Porter Stemming Algorithm [30]. For example, if “metabolism” was mentioned in a sentence, the root form “metabole” and common variations of “metabole”, including “metabolic”, “metabolite”, and “metabolize”, were replaced to create new sentences.

If mentions comprised multiple words, we connected each word using an underscore symbol, thus generating a single word. For example, if the mention “breast cancer” was identified from a sentence, a new sentence was created in which “breast cancer” was replaced by the single word “breast_cancer”. In addition, mentions that were not included in the training data cannot be represented as vectors. To increase the coverage of entities to be represented in the vector space, disease or plant names and their synonyms in the entity dictionary that were not included in the training data were added to the training data.

#### Word representations

Mikolov et al. developed Word2Vec [29], a neural network approach for computing the vector representations of words. Vectors can be constructed using two algorithms: a continuous bag-of-word (CBOW) model and a skip-gram model. The CBOW model learns word representations by predicting a word in a sentence using its surrounding words, and the skip-gram model learns word representations by predicting the surrounding words of a word in the input layer. In Word2Vec, words are represented by vectors in hundreds of dimensions, and words that have related meanings are more likely to have similar values in the vector space. A vector *w*_*t*_ for a word located at the *t*-th position in a sentence is calculated by maximizing the average log probability as follows:

- CBOW equation:
  1 $$ \frac{1}{T}\sum_{t=1}^{T} \text{log}~p\left(w_{t}|w_{t-\frac{c}{2}},{\ldots},w_{t-1},w_{t+1},{\ldots},w_{t+\frac{c}{2}}\right),  $$
- Skip-gram equation:
  2 $$ \frac{1}{T}\sum_{t=1}^{T} \text{log}~p\left(w_{t-\frac{c}{2}},{\ldots},w_{t-1},w_{t+1},{\ldots},w_{t+\frac{c}{2}}|w_{t}\right),  $$

where $w_{t-\frac {c}{2}},\ldots,w_{t-1},w_{t+1},\ldots,$ and $w_{t+\frac {c}{2}}$ are vectors for the surrounding *c* words in the sentence, and *T* is the number of tokens. We applied several options of a vector size of a word and a window size for surrounding words for both CBOW and skip-gram algorithms to train the models, and then, we chose the best options using the development sets.

To use unlabeled data in PubMed, we collected four groups of texts: (1) all PubMed abstracts (hereafter referred to as “all abstracts”), (2) biological-entity-specific abstracts that contain at least one biological entity name in the abstracts (“entity-specific abstracts”), (3) sentences that include at least one biological entity name in the sentence (“evidence sentences”), and (4) a collection of “evidence sentences” and modified evidence sentences (“modified evidence sentences”). Here, biological entities were identified using NER tools. For disease names, we used BANNER [27] because it has been used in several disease name recognition systems including DNorm and in several studies [17, 18, 31]. For plants, we applied LingPipe using exact matching based on the plant dictionary because several systems have used dictionary-based approaches for plant or species name recognition [32, 33]. Note that the NER systems were used to construct unlabeled data because the amount of unlabeled data is too large to manually curate entity names. Modified evidence sentences were constructed by replacing mentions of biological entities with concepts in the training set and synonyms in the dictionary as described in the “Incorporating information in training data sets” section. For example, “Van der Woude syndrome” is abbreviated as “VWS” and has a synonym of “lip pits”. Thus, a sentence in the trainning data “Affected males and females are equally likely to transmit VWS. (PubMed ID: 4019732)” generates following modified sentences:

1. “Affected males and females are equally likely to transmit Van der Woude syndrome”,
2. “Affected males and females are equally likely to transmit Van_der_Woude_syndrome”,
3. “Affected males and females are equally likely to transmit lip pits”, and
4. “Affected males and females are equally likely to transmit lip_pits”.

We propose four semi-supervised learning models. Each model constructs a vector set *V* of words representing words in the vector space by applying Word2Vec [29] to the training corpus and unlabeled data sets: (1) semi-supervised learning with unlabeled data of “all abstracts” (hereafter referred to as “SSL-all abstracts”), (2) semi-supervised learning with unlabeled data of “entity-specific abstracts” (“SSL-entity abstracts”), (3) semi-supervised learning with unlabeled data of “evidence sentences” (“SSL-evidences”), and (4) semi-supervised learning with unlabeled data of “modified evidence sentences” (“SSL-modified evidences”). In addition to these four models, we constructed (5) semi-supervised model that used only modified evidence sentences without the training corpus (“SSL-only modified evidences”). For comparison, we also constructed a supervised learning model with the training corpus (“SL-only training data”).

### Prediction for normalizing biological entities

As shown in Fig. 2, in the test step, abstracts in the NCBI disease corpus and in the plant corpus were used to test the normalization model. Biological mentions were extracted from the abstracts. If an extracted mention was exactly matched to a concept name, it was assigned to a corresponding concept ID, and additional normalization steps were not performed. Next, we applied an abbreviation resolution step, in which acronyms were changed to the original long words by using the abbreviation dictionary. The abbreviation resolution step is indicated by a dashed square because we investigated our proposed tool with and without the abbreviation step. For plants, we did not use the abbreviation step.

For the normalization, test mentions are mapped to their concepts by calculating the cosine similarities between a vector of the test mention and vectors of every possible concept in the entity dictionary. Then, words with high cosine similarities were considered candidate concepts (Fig. 2). Let a mention *m* and a candidate concept *c* be represented vectors v _*m*_ and v _*c*_, respectively. When a mention *m* comprises a single token such as “cancer” or “tumours”, a vector for the single token in the vector set *V* is assigned to v _*m*_. When a mention *m* comprises multiple tokens, v _*m*_ is assigned as the average of vectors for tokens in the mention as follows:

3 $$ \mathrm{v}_{m} = \frac{1}{n}\sum_{i=1}^{n} \mathrm{v}_{m_{i}},  $$

where v$_{m_{i}}$ is the vector of the *i*-th token in the mention and *n*, the number of tokens. If the *j*-th term vector v$_{m_{j}} \notin V$, we assign a zero vector to v$_{m_{j}}$ and calculate the average vector v _*m*_ by using Eq. (3). Note that concepts with multiple tokens were converted into a single token using an underscore symbol in the training step. After the mentions for biological entities were represented as vectors, concepts with high cosine similarities in word vectors v _*c*_∈*V* to the vector v _*m*_ of a query biological entity were recommended as normalized concepts.

#### Evaluation metric

To measure the performance of the disease name normalization tools, we compared highly ranked predicted concepts with manually mapped concepts in the test corpus. Table 2 shows an example of normalized disease names from the NCBI test set. “C7 defects” is the synonym of “COMPLEMENT COMPONENT 7 DEFICIENCY” as a disease mention in the NCBI disease test corpus, and the corresponding concept identifier is “OMIM:610102”. For a given mention, other names were ranked according to cosine similarities with the mention in the vector representation. Because a concept identifier includes several disease synonyms, asterisks in the first column indicate that these words are synonyms for the concept identifier, meaning that they are correctly recommended answers. In Table 2, the candidate mentions ranked first, second, third, fourth, fifth, sixth, and tenth are the correct results.

**Table 2:** An example of candidate normalized disease names for the mention “C7 defects”

	Ranks	Candidate names	Cosine
			similarity
∗	1	COMPLEMENT_COMPONENT_7_DEFICIENCY	0.559244
∗	2	complement_compon_7_defici	0.554464
∗	3	c7_defici	0.549911
∗	4	complement_component_7_deficiency	0.540654
∗	5	C7_DEFICIENCY	0.533657
∗	6	c7_deficiency	0.525014
	7	antibodi_defici_syndrom	0.510718
	8	Immunologic_Deficiency_Syndromes	0.499981
	9	immunolog_defici_syndrom	0.492753
∗	10	c7d	0.491925

The concept id of “C7 defects” is “OMIM:610102”, and the asterisk mark (∗) in the first column indicates that candidate names belong to “OMIM:610102”

**Fig. 3:**
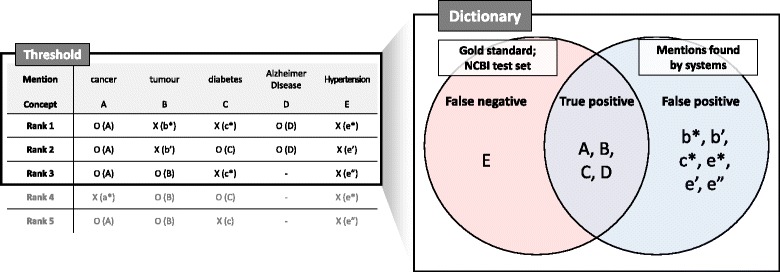
An example of lists of candidate concepts and accuracies. When the rank threshold is third, we consider concepts ranked from first to third as positives. “O” indicates that the predicted concepts are correct and “X” indicates that they are incorrect

We measured the performance of the normalization model for all mentions in the test set for each rank threshold. For the given rank threshold, the predicted names (or their corresponding concept IDs) that ranked higher than the threshold were considered positively predicted. True positives (*TP*) were correct positive predictions, false positives (*FP*) were incorrect positive predictions, and false negatives (*FN*) were mentions that are not positively predicted. For the case in which an extracted mention was exactly matched to a concept name, only a single concept ID was assigned, and it was a correct normalization. Therefore, when calculating the performance for each rank threshold, this exact match was treated as a true positive. Figure 3 shows an example of the candidate lists and *TP*, *FP*, and *FN*. The precision (*p*), recall (*r*), and F-score (*f*) are calculated as follows:

4 $$ p=\frac{TP}{TP+FP} \quad r=\frac{TP}{TP+FN} \quad f=\frac{2*p*r}{p+r}  $$

## Results

### Disease name normalization

To measure the performance of disease name normalization tools using the test corpus, we first extracted disease mentions in the 100 test abstracts using BANNER [27], and then, we manually curated correct disease mentions, thereby generating 843 test mentions. Note that because DNorm applied BANNER to extract candidate disease mentions from test abstracts, we also applied BANNER to compare normalization results under the same condition as DNorm.

For disease name normalization, we constructed six models: (1) “SSL-all abstracts” with 1,167,886 word vectors from 13,408,565 PubMed abstracts, (2) “SSL-entity abstracts” with 756,089 word vectors from 7,980,370 disease-related “entity-specific abstracts”, (3) “SSL-evidences” with 350,011 word vectors from 4,758,992 disease-related “evidence sentences”, (4) “SSL-modified evidences” with 740,353 word vectors, (5) “SSL-only modified evidences” with 714,575 word vectors, and (6) “SL-only training data” with 51,619 word vectors from the 592 NCBI disease training corpus.

Table 3 shows the comparison results of the four semi-supervised models, which combine training data and unlabeled data, for normalizing 843 disease mentions. To construct the word vectors used in the models, we used the default parameter values for the CBOW algorithm in Word2Vec: window size = 8 and vector dimension = 200. When “SSL-all abstracts” was used, the precision and F-score were the lowest. However, the model’s performance was similar to that of “SSL-evidences” and “SSL-entity abstracts”. Although more unlabeled data may increase the model performance in general, the results show that unlabeled data that are more relevant to entities led to slightly better results. “SSL-modified evidences” was the most powerful normalization tool, showing that the direct incorporation of entity synonyms in unlabeled data improved the normalization performance.

**Table 3:** Comparison of F-score of our disease normalization models using four biomedical text groups

Models	Win	Dim	Method	Precision	Recall	F-score
SSL-all abstracts	8	200	CBOW	0.627	0.832	0.715
SSL-entity abstracts	8	200	CBOW	0.633	0.838	0.721
SSL-evidences	8	200	CBOW	0.633	0.840	0.722
SSL-modified evidences	8	200	CBOW	**0.706**	**0.891**	**0.788**

The bold font denotes the best result for each column

Next, to find the optimal hyperparameters to learn word vectors, we applied different hyperparameters to the “SSL-modified evidences” model. When the NCBI disease development set was used to select hyperparameters, window size = 5 and vector dimension = 300, and a skip-gram method were selected (Table 4). The performance of the test set with these parameters was also close to the highest performance. Thus, these values were used in the following comparison.

**Table 4:** Performance comparison of disease normalization models using various parameters

				Development set			Test set		
Parameters	Win	Dim	Method	Precision	Recall	F-score	Precision	Recall	F-score
5_200_CBOW	5	200	CBOW	0.740	0.918	0.819	0.684	0.896	0.776
5_200_skip	5	200	Skip-gram	0.730	0.909	0.809	0.719	0.890	0.795
5_300_CBOW	5	300	CBOW	0.738	0.918	0.818	0.674	0.892	0.767
5_300_skip	5	300	Skip-gram	**0.746**	0.916	**0.822**	0.722	0.893	0.798
5_400_CBOW	5	400	CBOW	0.730	0.918	0.813	0.661	0.878	0.754
5_400_skip	5	400	Skip-gram	0.732	0.916	0.813	0.730	**0.905**	**0.808**
7_200_CBOW	7	200	CBOW	0.738	**0.919**	0.819	0.676	0.891	0.769
7_200_skip	7	200	Skip-gram	0.719	0.900	0.799	0.698	0.882	0.780
7_300_CBOW	7	300	CBOW	0.709	0.911	0.798	0.662	0.880	0.756
7_300_skip	7	300	Skip-gram	0.683	0.895	0.775	**0.776**	0.769	0.772
7_400_CBOW	7	400	CBOW	0.702	0.898	0.788	0.632	0.850	0.725
7_400_skip	7	400	Skip-gram	0.690	0.896	0.779	0.667	0.887	0.761
8_200_CBOW	8	200	CBOW	0.710	0.907	0.797	0.706	0.891	0.788

The bold font denotes the best result for each column

**Fig. 4:**
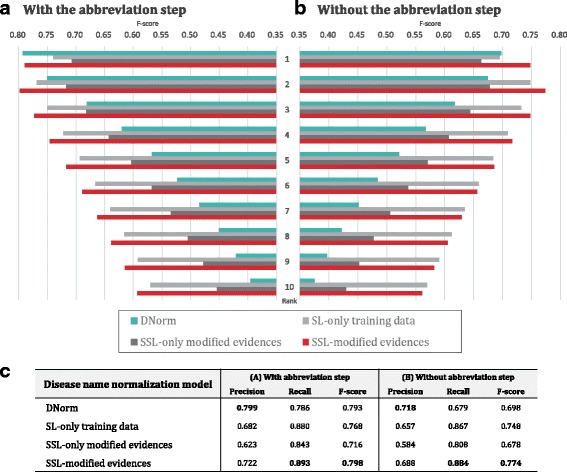
Performance comparison between DNorm and our models for disease name normalization with and without the abbreviation resolution step. In (**a**) and (**b**), dark-aqua bars indicate “DNorm” and the gray, dark-gray, and red bars indicate the “SL-only training data”, “SSL-only modified evidences” and “SSL-modified evidences” models, respectively. The *x*-axis represents the thresholds for ranks, and the *y*-axis indicates the F-scores of the models for each rank. **c** The precision, recall, and F-scores are shown for the four models

Moreover, we compared “SSL-modified evidences” with two additional cases: (1) “SL-only training data” and (2) “SSL-only modified evidences” with 714,575 word vectors. In addition, we compared DNorm [17] with our approach. Figure 4 shows performance comparisons with and without the abbreviation step. “SL-only training data” was better than “SSL-only modified evidences”, although “SSL-modified evidences” outperformed both cases. The results show that the normalization accuracies were improved when unlabeled data were incorporated with training data. The accuracy of “SSL-modified evidences” showed the best performance. Although the performance of our model was slightly higher than that of DNorm with the abbreviation step, it significantly outperformed DNorm without the abbreviation step. For DNorm, the F-score decreased significantly from 0.747 to 0.656 without the abbreviation step.

### Plant name normalization

For plant name normalization, we constructed three plant models: (1) “SL-only plant training data” with 94,338 word vectors, (2) “SSL-only modified plant evidences” with 594,802 word vectors, and (3) “SSL-modified plant evidences” with 649,759 word vectors. For plant evidence sentences, we collected 2,620,684 sentences containing plant names in the NCBI taxonomy database from PubMed abstracts. Note that because “SSL-modified evidences” showed the best performance for disease name normalization, we tested “SSL-modified plant evidences” among the several SSL models.

For selecting proper hyperparameters, we constructed the “SSL-modified plant evidences” model by applying different hyperparameters to the plant development set. Table 5 shows a comparison of several hyperparameters. We selected the hyperparameters as window size = 7 and vector dimension = 200, and we used the CBOW method.

**Table 5:** Performance comparison of plant normalization models using various parameters

				Development set			Test set		
Parameters	Win	Dim	Method	Precision	Recall	F-score	Precision	Recall	F-score
5_200_CBOW	5	200	CBOW	0.7284	0.8939	0.8027	**0.594**	**0.824**	**0.690**
5_200_skip	5	200	Skip-gram	0.6821	0.8812	0.7690	0.524	0.783	0.628
5_300_CBOW	5	300	CBOW	0.7326	0.8934	0.8051	0.576	0.811	0.674
5_300_skip	5	300	Skip-gram	0.6836	0.8813	0.7699	0.533	0.787	0.635
5_400_CBOW	5	400	CBOW	0.7311	**0.8940**	0.8044	0.568	0.809	0.667
5_400_skip	5	400	Skip-gram	0.7164	0.8878	0.7929	0.540	0.790	0.642
7_200_CBOW	7	200	CBOW	**0.7331**	0.8934	**0.8054**	0.590	0.822	0.687
7_200_skip	7	200	Skip-gram	0.7062	0.8840	0.7852	0.521	0.774	0.623
7_300_CBOW	7	300	CBOW	0.7320	0.8933	0.8047	0.589	0.818	0.685
7_300_skip	7	300	Skip-gram	0.7067	0.8833	0.7852	0.528	0.781	0.630
7_400_CBOW	7	400	CBOW	0.7163	0.8862	0.7922	0.554	0.798	0.654
7_400_skip	7	400	Skip-gram	0.6218	0.8859	0.7706	0.525	0.786	0.629

The bold font denotes the best result for each column

**Fig. 5:**
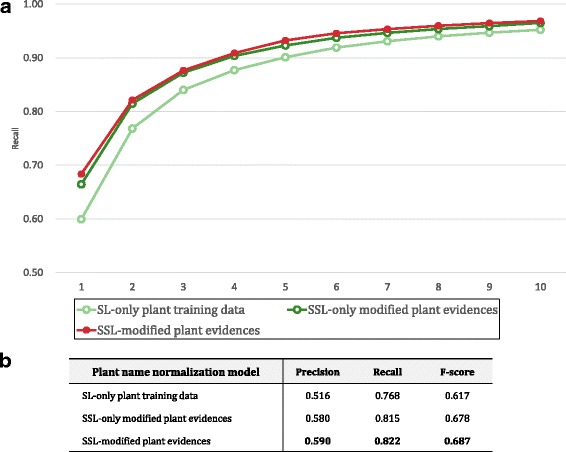
Performance comparisons of the proposed models for plant name normalization without the abbreviation resolution step. In (**a**), the light-green, dark-green, and red lines indicate the “SL-only plant training data”, “SSL-only modified plant evidences”, and “SSL-modified plant evidences” models, respectively. The *x*-axis represents the thresholds for ranks, and the *y*-axis indicates the recall of models for each rank. **b** The precision, recall, and F-scores are shown for the three models for plant name normalization

We tested the models using the plant corpus, for which an abbreviation dictionary was not available. Figure 5 shows the normalization results of 629 plant mentions from the plant test corpus. For plant normalization, “SSL-modified plant evidences” showed the best performance. Unlike the disease normalization result, “SSL-only modified evidences” was better than “SL-only training data”. Because an abbreviation dictionary was not available and plant names are usually represented by several types of names depending on their context, region, or language, plant name normalization showed lower accuracy compared to disease name normalization.

## Discussion

In this study, we compared the proposed approach to DNorm for disease name normalization. In the BioCreative V challenge [10], DNorm was used as a baseline system in the disease named entity recognition and normalization (DNER) task, and the F-score was 0.806. Therefore, we further evaluated our approach using a data set in the DNER task. Because our approach contains only the normalization step, we assumed that we already knew the correct disease mentions in the test data set of the DNER task, and then, we measured the normalization performance. In the DNER task, Lee et al.’s approach [15] ranked first with an F-score of 0.865; their approach used dictionary-based normalization by using five dictionaries with priorities in the order of CDR development/training sets from a subset of the BioCreative V corpus, MEDIC, NCBI disease corpus, and MEDIC extension lexicon. When we re-evaluated their normalization approach after assuming that all disease names were correctly recognized, the F-score was 0.982. For the purpose of comparison, we used the same dictionaries, and then applied the “SSL-modified evidences” model with the following parameter values: window size = 5 and vector dimension = 300 for the skip-gram algorithm in Word2Vec. As a result, we obtained an F-score of 0.986. The performances of these two systems were similar with very high accuracies; this might be due to the high-quality dictionaries used, such as the CDR development/training sets and MEDIC. Therefore, after excluding dictionaries from the CDR development/training sets, MEDIC, and MEDIC extension lexicon and by using the NCBI disease corpus, we evaluated the two systems. Note that because we excluded MEDIC, we used the “SSL-evidences” model in this evaluation; training data was constructed using the NCBI disease corpus and unlabeled data was constructed using sentences containing disease names from PubMed. The F-scores of the dictionary-based approach and our approach reduced to 0.324 and 0.659, respectively. This shows the importance of high-quality dictionaries; at the same time, our system can achieve better performance even without good dictionaries.

In this study, we applied the Word2Vec algorithm for the word representation. In addition to Word2Vec, several studies for word representation have succeeded in capturing fine-grained semantic meanings. GloVe [34] is an alternative model for learning word embeddings. For comparison with Word2Vec, we generated word embeddings using the GloVe algorithm with different sets of parameters, and used them for the normalization. As a result, we obtained an F-score of 0.639 for disease name normalization using GloVe trained with the same parameters as Word2Vec as follows: window size = 5, vector dimension = 300, and iteration = 1000. Compared to Table 4, word embeddings obtained by Word2Vec outperformed those generated by Glove. Indeed, several studies showed that Word2Vec outperformed GloVe on word similarity tasks although GloVe achieved the best performance on the word analogical reasoning task [35, 36]. In addition to GloVe, Luong et al. [37] proposed morphological recursive neural networks (RNNs) that combine RNNs and neural language models to learn word embeddings from morphemes. Wang et al. [38] applied the bidirectional LSTM-RNN structure to represent word vectors, which outperformed CBOW and skip-gram approaches in Word2Vec when tested for the NER task. Thus, in the future work, we will improve the normalization performance by enhancing a neural network architecture for word representation.

## Conclusions

In this study, we integrated training data and unlabeled data for word representation in entity name normalization and verified that the proposed normalization model is a useful tool for disease names and plant names. For many biological entities, there is no comprehensive dictionary; therefore, our approach will be useful for normalizing various entities.

## Additional file

Additional file 1 Guidelines of our plant corpus. (PDF 177 kb)

## Abbreviations

All abstracts: All PubMed abstracts
AR: Androgen receptor
AS: Angelman syndrome
AS: ankylosing spondylitis
BioCreative: The Critical Assessment of Information Extraction in Biology
C7 defects: Complement component 7 deficiency
CBOW: Continuous bag-of-word
Entity-specific abstracts: Biological-entity-specific abstracts that contain at least one biological entity name in the abstracts
Evidence sentences: sentences that include at least one biological entity name in the sentence
f: F-score
FN: False negatives
FP: False positives
IAA: Inter-annotator agreement
IDs: Identifiers
LSTM: Long short-term memory
MEDIC: Merged disease vocabulary
MeSH: Medical subject headings
ML: Machine learning
Modified evidence sentences: A collection of “evidence sentences” and modified evidence sentences
NCBI: National center for biotechnology information
NER: Named entity recognition
NLP: Natural language processing
OMIM: Online mendelian inheritance in man
p: Precision
PCa: Prostate cancer
r: Recall
RNNs: Recursive neural networks
TP: True positives
SL-only training data: Supervised learning model with the training corpus
SSL-all abstracts: Semi-supervised learning with unlabeled data of “all abstracts”
SSL-entity abstracts: Semi-supervised learning with unlabeled data of “entity-specific abstracts”
SSL-evidences: Semi-supervised learning with unlabeled data of “evidence sentences”
SSL-modified evidences: Semi-supervised learning with unlabeled data of “modified evidence sentences”
SSL-only modified evidences: Semi-supervised model that used only modified evidence sentences without the training corpus
UMLS: Unified medical language system
VWS: Van der Woude syndrome
