# Supplementary material for: A method for named entity normalization in biomedical articles: application to diseases and plants
Source: BMC Bioinformatics. 2017 Oct 13;18:451. doi: 10.1186/s12859-017-1857-8 (PMC5640957; doi:10.1186/s12859-017-1857-8)
Supplement: Additional file 1 — Guidelines of our plant corpus. (PDF 177 kb) [file 12859_2017_1857_MOESM1_ESM.pdf]

Construction of the plant corpus

Selection of abstracts

For the selection of candidate abstracts that will be annotated by two curators, we extracted 13,408,621 PubMed abstracts using PubTator [1]. Then, pre-processing steps were performed as follows:

1 For the annotation of plant names in abstracts, it is necessary to identify an entity mention with its identifier and the offset of the location of each entity mention in texts. Thus, we used a well-known named entity recognition tool because it is hard to manually annotate all plant mentions in whole abstracts. For annotating plant names, we applied Lingpipe [2] to PubMed abstracts for locating plant names using NCBI taxonomy [3].

2 As a result of the first step, we obtained 773,312 abstracts and then randomly selected 208 candidate abstracts to construct the plant corpus.

3 When performing annotation tasks, we applied a BRAT system [4] that is a web-based tool designed for text annotation.

Curator guidelines

The guidelines for curators were designed to annotate all strings that can be identified as plant names. We performed manual annotation of all plant mentions from 208 abstracts. For each entity occurrence, we annotated its text span and assigned a corresponding concept identifier from NCBI taxonomy [3]. Although the main focus of annotation is to annotate plant mentions, low taxonomic levels (e.g. family, tribe, genus, species) were also considered. The guideline for plant mentions consists of two following sub-sections.

1 Annotation guideline for plant:
- All plant mentions including repetitions should be listed in the order of appearance in the abstracts.
- The annotated mentions are composed of Linnaean plant names, general names, scientific names, and author defined acronyms.
- The annotated mentions might have any misspellings (or other typing errors) in the articles. (e.g. *mari**j**uana* and *mari**h**uana*)
- If there is a compound word composed of a plant name and any noun, a plant mention that represents the actual contextual meaning should be annotated. (e.g. ***olive*** *groves* and ***sorghum*** *genome*)
- Although a compound word contains a plant name, the name should not be annotated if its meaning is not botanical. (e.g. ***melon*** *fly* and ***cinnamon*** *rat*)

2 Normalization guideline for plant:
- NCBI taxonomy identifiers [3] are used for plant identifiers.
- If a plant mention has more than one identifier, annotators should annotate all identifiers by distinguish them with a "|" symbol. (e.g. *maize* **4577**|**381124**)
- When an annotator cannot find a proper identifier of the plant mention from the taxonomy dictionary, the annotator should search other synonyms using the Google search engine.

- When an annotator cannot search a proper identifier even using Google, the annotator should annotate their botanical low-level concepts (from Family to lowest) using Wikipedia (e.g. Gynura cusimbua {Family-Tribe-Genus: Asteraceae-Senecioneae-Gynura Cass.} [**4210-102812-109564**])
- When an annotator cannot find a proper identifier anymore, the plant identifier should be annotated as "NA."

Inter-annotator Agreement (IAA)

For plant entity annotation, we recruited two curators who have previous experience for annotation. We used a Jaccard coefficient score to measure an IAA score that represents the consistency of annotations. A_1 and A_2 indicate the set of annotated results by the first and second annotators, respectively. Annotated mentions having the same PMID, start and end points, and taxonomy identifiers were considered as the agreement cases and others were counted as the disagreement cases. We measured mention-level and normalization-level accuracies. For example, if both A_1 and A_2 contain the "rosemary," it was counted as a mention-level agreement. If A_1 contains "Silkbay and camphor tree," and A_2 separately contains "Silkbay" and "camphor tree," this case was counted as disagreements in the mention-level. For the normalization-level measurement, we applied the same approach in terms of identifiers. Therefore, we can measure the Jaccard index Jac_{A_1,A_2} by counting the number of agreements as follows.

$$Jac_{A_1,A_2} = \frac{|A_1 \cap A_2|}{|A_1 \cup A_2|} \quad (1)$$

Result

IAA analysis

An IAA score of the mention-level was obtained by considering correct identification of plant mentions regardless of labels of the taxonomy identifiers. For an IAA score of normalization-level, both plant mentions and identifiers of plant mentions should be correctly annotated. Table 1 shows overall IAA scores for annotations.

Table 1 IAA anaylsis

Annotators	# of abstracts	# of annotated results	$A_1 \cup A_2$
A_1	208	3985	3997
A_2		3983	
**Type**	**$A_1 \cap A_2$**	**Jaccard index**	
Mention	3936	Jac_{A_1,A_2}(mention)	**0.985**
Normalization	3556	Jac_{A_1,A_2}(normalization)	**0.890**

Disagreement and harmonization

We compared annotations from two curators to harmonize the annotated results. For the mention-level annotation, 61 out of 3,997 candidate mentions were disagreed between annotators with the following cases; one annotator incorrectly annotated (six false positives, 9.8%), one annotator did not annotate an actual plant mention (17 false negatives, 27.9%), and one annotator partially annotated (38 cases, 62.3%). We introduce examples of the discrepant cases and resolution of disagreements.

- **Example 1.** Comparative study of the assay of [Artemia salina L.]$_{A_1}$ and the estimate of the medium lethal dose (LD50 value) in mice, to determine oral acute toxicity of plant extracts. [PMID:11695884]
  - For the sentence in Example 1, one annotator assigned the annotated mention as a plant. However, the mention "Artemia salina L." indicates a species of brine shrimp. Thus, we omit this mention from our plant corpus according to the guidelines.
- **Example 2.** The effect of photosynthetic types on xylene removal efficiency suggests that a mixture of [Z. zamiifolia]$_{A_1,A_2}$, [S. hyacinthoides]$_{A_1,A_2}$, and [A. commutatum]$_{A_1}$ which represent facultative CAM, CAM, and C3 plants, is the most suitable system for xylene removal. [PMID:24091527]
  - For the sentence in Example 2, one annotator failed to assign "A. commutatum" as a plant mention. Actually, "A. commutatum" is used as abbreviation for "Aglaonema commutatum" in the abstract. Thus, the mention was added to the plant corpus after two annotators discussed it.
- **Example 3.** [[Silkbay]$_{A_1}$ and [camphor tree]$_{A_1}$]$_{A_2}$ attracted the highest numbers of the beetle in the field, and [lancewood]$_{A_1,A_2}$ and [spicebush]$_{A_1,A_2}$ the lowest, whereas boring activity was greatest on [silkbay]$_{A_1,A_2}$, [bay laurel]$_{A_1,A_2}$, [swampbay]$_{A_1,A_2}$, and [redbay]$_{A_1,A_2}$, and lowest on [lancewood]$_{A_1,A_2}$, [spicebush]$_{A_1,A_2}$, and [camphor tree]$_{A_1,A_2}$. [PMID:25007073]
  - For the sentence in Example 3, one annotator assigned "Silkbay and camphor tree" as a plant mention while the other annotator annotated "Silkbay" and "camphor tree" as two separate plant names. Because "Silkbay" and "camphor tree" are different plants, "Silkbay and camphor tree" was separately annotated as two plant mentions: "Silkbay" and "camphor tree."

For the normalization-level annotation, 441 annotations were in disagreement. The disagreements were largely divided into the following three cases: 26 unmapped identifiers (5.9%), 261 partially matched identifiers (59.2%), and 154 mismatches (34.9%). Two annotators resolved disagreement by discussion and reached on agreement for all plant corpus. As a result, Table 2 shows details of the plant corpus.

Table 2 Plant corpus

Data Sets	Abstracts	All plant mentions	Unique plant mentions	Unique concept IDs
Plant training set	128	2,647	1,543	1,143
Plant development set	40	709	400	329
Plant test set	40	629	427	298
Total	208	3,985	2,370	1,770

Author details

References

1. Choi, W., Lee, H.: A text mining approach for identifying herb-chemical relationships from biomedical articles. In: Proceedings of the ACM Ninth International Workshop on Data and Text Mining in Biomedical Informatics, pp. 25–25 (2015). ACM
2. Carpenter, B.: Lingpipe for 99.99% recall of gene mentions. In: Proceedings of the Second BioCreative Challenge Evaluation Workshop, vol. 23, pp. 307–309 (2007)
3. Federhen, S.: The ncbi taxonomy database. Nucleic acids research **40**(D1), 136–143 (2012)
4. Stenetorp, P., Pyysalo, S., Topić, G., Ohta, T., Ananiadou, S., Tsujii, J.: Brat: a web-based tool for nlp-assisted text annotation. In: Proceedings of the Demonstrations at the 13th Conference of the European Chapter of the Association for Computational Linguistics, pp. 102–107 (2012). Association for Computational Linguistics
